# Supplementary material for: Precision Medicine: Determination of Ribavirin Urinary Metabolites in Relation to Drug Adverse Effects in HCV Patients
Source: Int J Mol Sci. 2022 Sep 2;23(17):10043. doi: 10.3390/ijms231710043 (PMC9456413; doi:10.3390/ijms231710043)
Supplement: Supplementary file 1 [file ijms-23-10043-s001.zip › ijms-1844313-supplementary.pdf]

Precision Medicine: Determination of Ribavirin Urinary Metabolites in Relation to Drug Adverse Effects in HCV Patients

Ottavia Giampaoli [1,2,†], Fabio Sciubba [1,2,†], Elisa Biliotti [3], Mariangela Spagnoli [4], Riccardo Calvani [5], Alberta Tomassini [1,6], Giorgio Capuani [1,6], Alfredo Miccheli [1,2,*] and Gloria Taliani [3]

[1] NMR-Based Metabolomics Laboratory (NMLab), Sapienza University of Rome, Rome, 00185, Italy;
ottavia.giampaoli@uniroma1.it (O.G.); fabio.sciubba@uniroma1.it (F.S.);
alberta.tomassini@uniroma1.it (A.T.); giorgio.capuani@uniroma1.it (G.C.)
[2] Department of Environmental Biology, Sapienza University of Rome, Rome, 00185, Italy
[3] Department of Clinical Medicine, Policlinico Umberto I, Sapienza University of Rome, Rome, 00161, Italy;
elisa.biliotti@uniroma1.it (E.B.); gloria.taliani@uniroma1.it (G.T.)
[4] Department of Occupational Medicine, Epidemiology and Hygiene, INAIL, Monte Porzio Catone, Rome, 00078, Italy; m.spagnoli@inail.it
[5] Fondazione Policlinico Universitario A. Gemelli IRCCS, Rome, 00168, Italy; riccardo.calvani@gmail.com
[6] Department of Chemistry, Sapienza University of Rome, Rome, 00185, Italy
* Correspondence: alfredo.miccheli@uniroma1.it
† These authors contributed equally to this work.

SUPPLEMENTARY MATERIALS

Table S1. Characteristics of the enrolled patients at baseline, at treatment week 4 (TW4) and at the end of treatment (EOT).

	**Baseline (T0)**	**Treatment Week 4 (TW4)**	**End of Treatment (EOT)**
**Age, years - mean ± SD**	58.41 ± 8.80	-	-
**Male gender – number (%)**	17 (100%)	-	-
**BMI, kg/m^2- mean ± SD**	25.03 ± 3.64	-	-
**GOT, U/L - mean ± SD**	93.29 ± 62.68	24.38 ± 8.68	24.30 ± 9.86
**GPT, U/L - mean ± SD**	118.12 ± 86.89	24.19 ± 8.65	22.29 ± 9.92
**γGT, U/L - mean ± SD**	120.88 ± 88.11	40.13 ± 17.29	28.29 ± 17.07
**e-GFR, ml/min/1.73m^2 - mean ± SD**	94.99 ± 7.05	91.90 ± 10.06	91.64 ± 11.84
**HCV genotype, number (%)**			
1a	13 (76.47%)	-	-
1b	4 (23.53%)	-	-

Table S2. Interferon free regimens for HCV treatment. SOF: Sofosbuvir ; LDV: Ledipasvir; SMV: Simeprevir; 3D: Paritaprevir/Ritonavir, Ombitasvir, Dasabuvir; RBV: Ribavirin

**Treatment regimens – number (%)**	
SOF-LDV<br>SOF-SMV<br>3D	7 (41.18%)<br>6 (35.29%)<br>4 (23.53%)
**Treatment regimens containing RBV – number (%)**	17 (100%)
**Treatment regimens containing SOF – number (%)**	13 (76.47%)

Table S3. Hemoglobin (Hb) values of patients at baseline (T0), TW4 and EOT stages. NA: not available.

Patient ID	Hb T0 (g/dl)	Hb TW4 (g/dl)	Hb EOT (g/dl)
**09**	16.6	12.8	13.4
**11**	16.7	13.3	13.4
**12**	16.1	12.1	13.4
**14**	15.9	13.3	13.9
**16**	16.2	13.8	13.2
**19**	17.4	15	14.4
**20**	15.4	12.7	13.2
**21**	15.4	13.2	13.7
**27**	15	13.3	13.6
**40**	15.2	12.8	12.8
**42**	13.3	11.8	11.6
**43**	NA	NA	NA
**44**	16.1	14.4	14.2
**45**	16.3	13.6	13.6
**47**	15.3	13.7	13.4
**48**	14	11.2	11.5
**50**	15.3	14.5	14.1

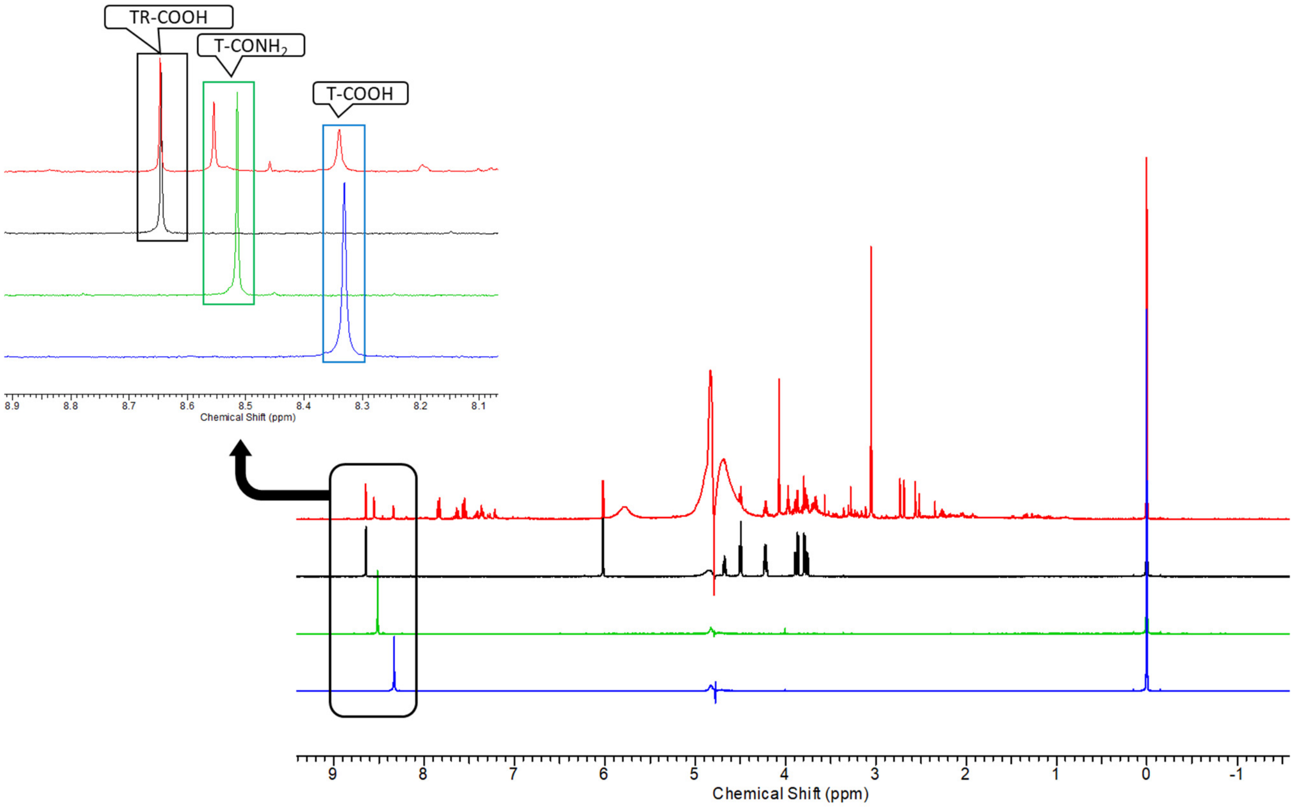

Figure S1. From the bottom to top, ^{1}H-NMR spectra of T-COOH (blue), T-CONH$_2$ (green), TR-COOH (black) and a control urine spiked (red) to each standard. Small variations of chemical shift can be attributed to different ionic strength and small variation of pH in urine sample.

We added to the urine TR-COOH at final concentration of 0.9 mM, T-CONH$_2$ at final concentration of 0.7 mM and T-COOH at final concentration of 0.8 mM. The chemical shifts are confirmed for the assigned molecules, also on the basis of the relative integrals.
